# Lipidomic analysis of serum samples from migraine patients

**DOI:** 10.1186/s12944-018-0665-0

**Published:** 2018-02-02

**Authors:** Caixia Ren, Jia Liu, Juntuo Zhou, Hui Liang, Yayun Wang, Yinping Sun, Bin Ma, Yuxin Yin

**Affiliations:** 1 Departments of Human Anatomy, Histology and Embryology, Peking University Health Science Center, Beijing, 100191 China; 2 Institute of Systems Biomedicine, Department of Pathology, School of Basic Medical Sciences, Peking University Health Science Center, Beijing, 100191 China; 3 Department of Neurology, Dongzhimen Hospital Affiliated to Beijing University of Chinese Medicine, Beijing, 100700 China; 4 Dongfang Hospital Affiliated to Beijing University of Chinese Medicine, Beijing, 100078 China; 5 Beijing Key Laboratory of Tumor Systems Biology, Peking-Tsinghua Center for Life Sciences, Beijing, 100191 China

**Keywords:** Migraine, Lipidomics, Biomarkers, LysoPC, Acylcarnitine, Cer_NS, LysoPE

## Abstract

**Background:**

Migraine is a prevalent, disabling type of primary headache disorder associated with a high socioeconomic burden. The clinical management of migraine is challenging. This study was to identify potential serum lipidomic biomarkers of migraine.

**Methods:**

The serum lipidomic profile of migraine sufferers was compared with healthy individuals using Liquid Chromatography coupled to Mass Spectrometry (LC-MS). Volcano plot analysis by Student’s t-test was performed to identify the differential metabolites. Receiver operating characteristic (ROC) curves were constructed and the area under ROC curves (AUC) was calculated to evaluate whether the metabolites could be efficiently exploited for constructing a sensitive biomarker of migraine.

**Results:**

A total of 29 serum metabolites from 4 classes of lipids including acylcarnitines, non-alpha-hydroxy-sphingosine ceramides (Cer_NSs), lysophosphatidylcholines (lysoPCs) and lysophosphatidylethanolamines (lysoPEs) were significantly different in migraine patients and controls. Of note, Cer_NSs were significantly elevated and lysoPEs were significantly decreased in migraine patients. LysoPE 18:1, lysoPE 18:2 and lysoPE 22:5 were found to be decreased in both positive and negative ion mode. Moreover, except for lysoPC 20:0, other lysoPCs were decreased in migraine patients. ROC curve analysis indicated that lysoPC 16:0 and lysoPC 20:0 are potential sensitive and specific biomarkers for migraine.

**Conclusion:**

LysoPC 16:0 and lysoPC 20:0 may be potential biomarkers for migraine. We suggest therapeutic management of these metabolites may be helpful in the prevention and treatment of migraine.

**Electronic supplementary material:**

The online version of this article (10.1186/s12944-018-0665-0) contains supplementary material, which is available to authorized users.

## Background

Migraine is a common headache disorder with a lifetime prevalence of 13% in men and 33% in women [1]. The clinical features of migraine include intense and pulsating head pain localized to one side of the head that can effectively disable its sufferers for up to 72 h, Accompanying symptoms of migraine in addition to the headache include nausea, vomiting, and hypersensitivity to lights, sounds and/or smells, all of which can be aggravated by physical activity [2]. Migraine is the 8th most burdensome disease in the world overall, and the 4th most burdensome disease in women according to the 2012 Global Burden of Disease study, as it is associated with poor health-related quality of life, missed work days, and a high economic burden [3]. Despite intensive research, the exact pathologic mechanism of migraine is still poorly understood, making the discovery of more effective treatments extremely difficult. Many medications are used to treat migraine, however, none of these pharmaceutical agents has shown universal treatment efficiency, and side effects often limit their use [4]. At the same time, recurrences of symptoms may require increased use of a given drug, which may lead to excessive use and dissatisfaction with available treatments [5]. Therefore, seeking for new potential biomarkers is very important to help prevent and treat migraine.

In addition to genomics and proteomics, lipidomics have been employed as a metabolomic approach for investigation of the qualitative and quantitative profiles of lipid metabolites from serum, plasma, tissue, cells and organisms [6]. Circulating lipid metabolites are associated with many physiologic and pathologic processes in the body, and may therefore be of use for selecting biomarkers for prevention and treatment of disease [7]. To the best of our knowledge, there are few reports on lipidomic profiling of migraine.

In this study, we aimed to examine serum lipidomic metabolites with Liquid Chromatography coupled to Mass Spectrometry (LC-MS) to identify potential migraine biomarkers which may provide useful fundamental information for the prevention and treatment of migraine.

## Methods

### Patients

Twenty patients with migraine without aura, and 20 healthy control subjects in whom migraine was excluded by examination participated in this study (Table 1). The control subjects were recruited from hospital and laboratory personnel, and were age and sex matched with the study patients. Migraine was diagnosed by physicians specialized in this area of medicine utilizing the current International Headache Society (IHS) criteria (ICHD-3, beta version currently available at https://www.ichd-3.org/) [2]. Subjects with hypertension, diabetes, high cholesterol, morbid obesity, history of cardiovascular events, chronic renal failure, liver cirrhosis, thyroid diseases, history of significant head trauma, malnutrition, pregnancy, cigarette smoking, alcohol and substance abuse, chronic neurologic illnesses, including epilepsy, Parkinson’s disease, Huntington’s disease, Alzheimer’s disease, Wilson’s disease were excluded from both groups. None of these patients were taking any medication except for treatment of migraine attacks. On the day of study, patients were all headache free for at least 5 days. These patients abstained from taking any medication until the end of the study period. All subjects gave informed consent for inclusion before they participated in the study. The study was conducted in accordance with the Declaration of Helsinki, and the protocol was approved by the Ethics Committee of the Beijing University of Chinese Medicine (ECPJ-BDY-2016-06) (Additional file 1: Figure S1).

**Table 1:** Baseline characteristics of the study populations (mean ± SD)

Characteristics	Control	Migraine
Numbers	20	20
Gender (Male/Female)	8/12	8/12
Age (year)	32.85 ± 7.47	31.85 ± 9.1

### Chemicals and reagents

Formic acid, HPLC grade methanol, acetonitrile (ACN) and isopropanol (IPA) were obtained from Fisher Scientific. Chloroform was obtained from Tong Guang Fine Chemicals Company (Beijing, China). Free fatty acid (FFA) 19:0 and ammonium acetate was purchased from Sigma-Aldrich (St. Louis, MO, USA). Ultra-pure water was supplied by a Millipore system (Millipore, Billerica, MA, USA).

### Sample preparations

Lipids were extracted from serum samples by a modified Folch method [8]. Typically, 100 μL of serum were aliquoted into a 0.6 ml Eppendorf tube and mixed with 400 μL of chloroform/methanol (2:1, *V*/V) containing 20 μg/ml of free fatty acid 19:0 as an internal standard. After vortexing for 10 min, the mixture was centrifuged at 13000 rpm at 4 °C for 20 min. The lower lipid containing chloroform phase was evaporated with a speed vacuum, and the residue was stored at − 80 °C for further analysis. All samples were processed in the same laboratory to avoid bias.

### High-performance liquid chromatography

An Ultimate 3000 ultra high performance liquid chromatography (UHPLC) system coupled to Q-Exactive MS (Thermo Scientific) was used for lipid separation and detection. Samples were reconstituted in 20 μL chloroform/methanol (1:1, *V*/V) and diluted three times in IPA/ACN/water (2:1:1, V/V/V). After centrifugation at 12000 rpm for 15 min, 5 μL of supernatant were injected for LC-MS/MS analysis.

Chromatographic separation was performed on a reversed phase X select CSH C18 column (4.6 mm × 100 mm, 2.5 μm, Waters, USA), which is consistent with what has been reported [9]. Two solvents were used for gradient elution: (A) ACN/water (3:2, V/V), (B) IPA/ACN (9:1, V/V). Both A and B contained 10 mM. ammonium acetate and 0.1% formic acid. The gradient program was: 0 min-40% B; 2 min-43% B; 2.1 min-50% B; 12 min 60% B; 12.1–75% B; 18 min-99% B; 19 min-99% B; 20 min-40% B; 25 min-40% B. The column temperature was maintained at 50 °C, and the flow rate was set to 0.6 ml/min.

### Mass spectrometry

Mass spectrometric detection was performed by electrospray ionization in both positive ion mode and negative ion mode. The source voltage was maintained at 3.3 kV in the positive ion mode and 2.8 kV in the negative ion mode. All other interface settings were identical for both positive ion mode and negative ion mode. The capillary temperature, sheath gas flow and auxiliary gas flow were set at 320 °C, 40 arb and 10 arb respectively. Data were collected in a data-dependent top 10 scan mode. Survey full-scan MS spectra (mass range *m/z* 80 to 1200) were acquired with resolution *R* = 70,000 and AGC target 1e6. MS/MS fragmentation was performed using high-energy c-trap dissociation (HCD) with resolution *R* = 17,500 and AGC target 1e5. The stepped normalized collision energy (NCE) was set to 15, 30, and 45 respectively. External mass calibration was applied before every sequence run.

The stability of retention time, mass accuracy and intensity is essential in LC-MS based lipidomic analysis. A pooled serum sample was therefore prepared as QC to assess the stability of the instrument and ensure the reliability of the data. A QC sample was run before and after the sequence and in every 10 sample runs in the sequence in order to ensure the reproducibility of the data.

### Data processing and analysis

The acquired raw data were processed using MSDIAL according to the instructions in the software tutorial [10]. Datasets containing m/z values, retention time, and peak area were exported as an Excel file, and then the Excel file was imported into the Metabo Analyst 3.0 Web service [11] for multivariate analysis. Principal component analysis (PCA) which is an unsupervised chemometric method, was used to obtain an overall picture of the whole data sets, and to see if there were any clustering, trends, or outliers. Orthogonal partial least squares-discriminant analysis (orthoPLS-DA) was used for further data analysis. Volcano plot analysis with fold-change (FC) > 1.5 and false discovery rate (FDR) < 0.05 by Student’s t-test was performed to identify the differential metabolites. ROC curves were constructed and the area under ROC curves (AUC) was calculated to investigate whether the characteristics of the metabolites that differed significantly between two groups could be efficiently exploited for constructing a sensitive biomarker of migraine status.

## Results

### Patient characteristics

A total of 20 migraine patients and 20 controls were recruited for screening and identification of promising metabolites (Table 1). The mean ages for migraine patients and controls were 31.85 ± 9.1 and 32.85 ± 7.47 years, respectively (*p* = 0.819).

The causes of migraine were showed in Table 2. Most of them (16/20) suffered from anxiety and insomnia; 2 of 16 cases were accompanied by physical stimuli such as light or smell; 4 of 16 were accompanied by dietary factors such as cold drink or coffee; 2 of 16 were accompanied by physical activities such as running or taking bus; and 2 of 16 were accompanied by weather changes such as wind or cold stimuli. The remaining 4 (4/20) migraine were resulted from physical stimuli such as light, noise or smell alone.

**Table 2:** Causes of migraine in this study

Causes of migraine	Numbers
Anxiety and insomnia alone	6
Anxiety and insomnia accompanied by:	
Physical stimuli such as light or smell	2
Dietary factors such as cold drink or coffee	4
Physical activities such as running or taking bus	2
Weather changes such as wind or cold stimuli	2
Physical stimuli such as light, noise or smell alone	4
Total	20

### Identification of potential metabolic biomarkers

**Fig. 1:**
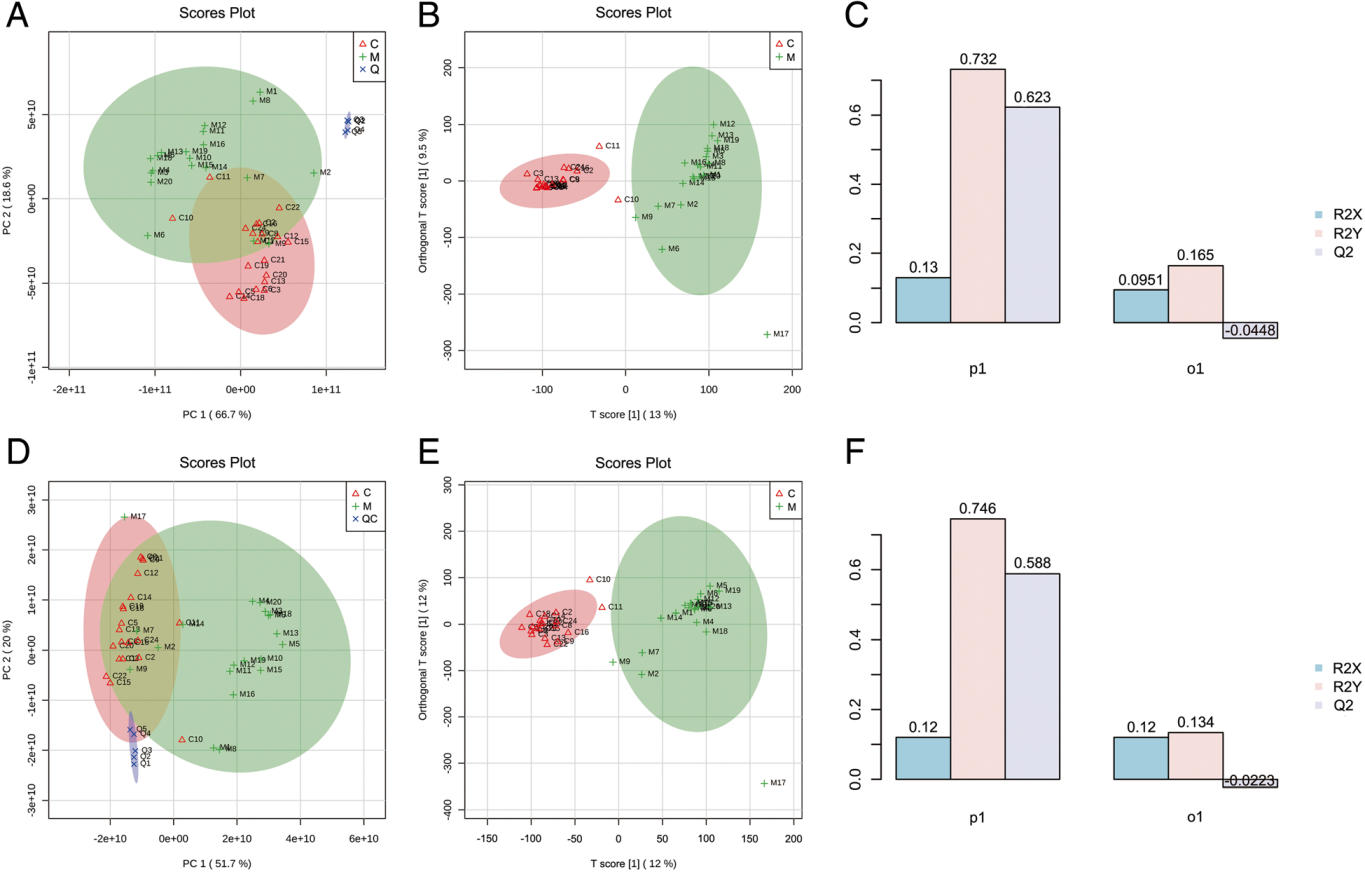
PCA (**a**) and orthoPLS-DA (**b**) score plots of migraine patients based on metabolomics data (positive ion mode). PCA (**d**) and orthoPLS-DA (**e**) score plots of migraine patients based on metabolomics data (negative ion mode). Performance statistics of orthoPLS-DA in positive ion mode (**c**) and negative ion mode (**f**)

PCA score plots which include migraine patients, healthy controls and quality control (QC) samples are shown in Fig. 1a (positive ion mode) and 1D (negative ion mode). All samples scattered in these figures fell into the 95% confidence interval. QC samples (blue) clustered together tightly, reflecting the stability of the LC-MS system and showing that the quality of all the LC-MS data for this study were satisfactory. The migraine group (M group, green) and healthy control group (C group, pink) were clustered together and separated from each other, despite a few overlapping data points in these two groups. This suggested that the involved metabolites were perturbed in the migraine group. In order to maximize the separation of the migraine and control groups, we found it was necessary to input a priori information about these two classes and use supervised models, such as Orthogonal partial least squares-discriminant analysis (orthoPLS-DA). The score plot in the orthoPLS-DA showed better clustering tendency between the migraine (green) and control groups (pink) as shown in Fig. 1b (positive ion mode) and Fig. 1e (negative ion mode). The percentage of extracted variance related to class information was 13% (positive ion mode) and 12% (negative ion mode) and explained 73.2% of the variance of class variables in positive ion mode (R^2^Y = 0.732)(Fig. 1c) and 74.6% of the variance of class variables in negative ion mode (R^2^Y = 0.746)(Fig. 1f). These multivariate analyses showed significant metabolic differences in the migraine and control groups.

**Fig. 2:**
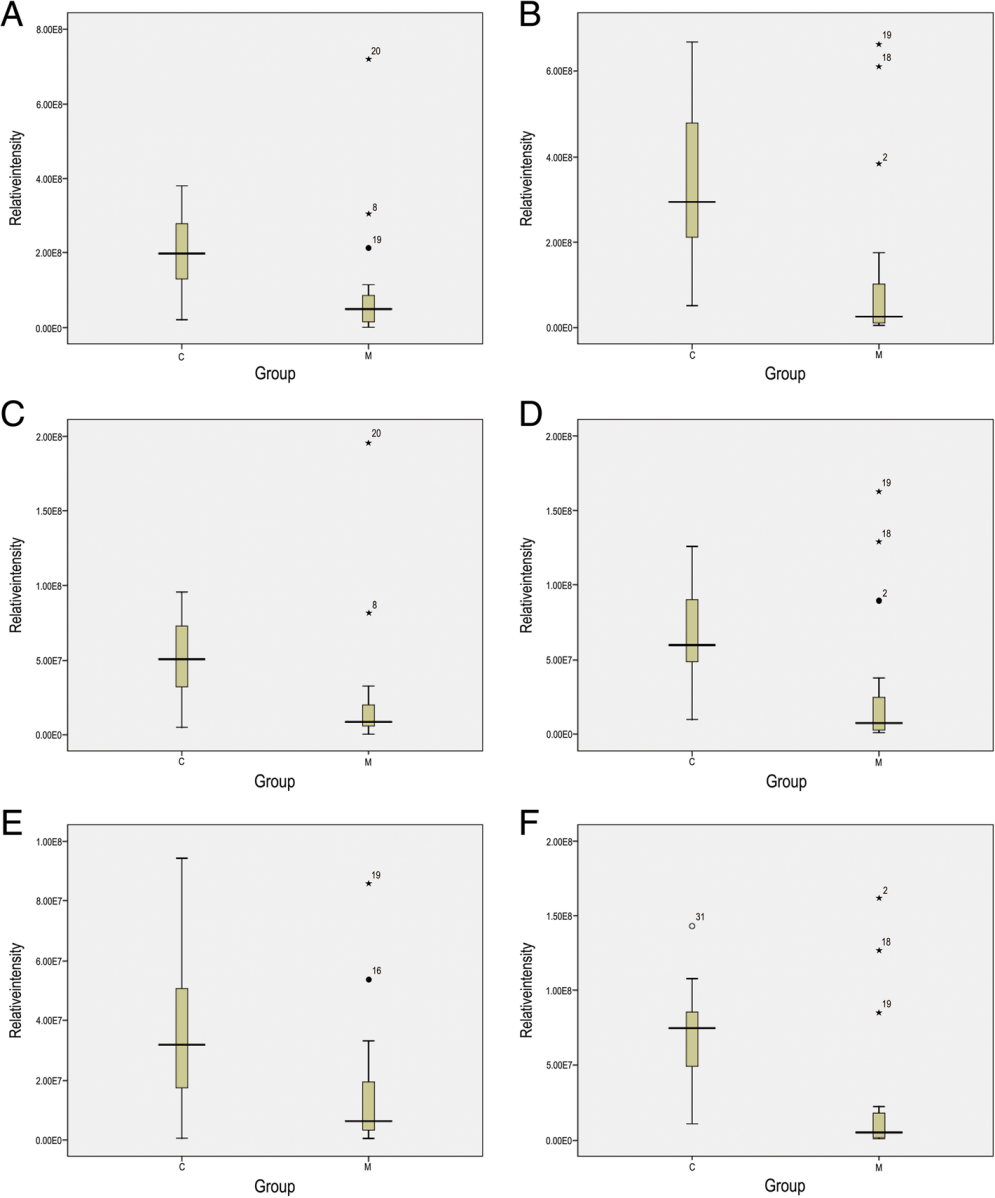
Boxplots representing relative abundance of: (**a**) lysoPE 18:2, [M + H]+; (**B**) lysoPE. 18:1, [M + H]+; (**C**) lysoPE22:5, [M + H]+; (**D**) lysoPE 18:2, [M-H]-; (**E**) lysoPE 18:1, [M-H]-; (**F**) lysoPE22:5, [M-H]-

A total of 1026 metabolites in positive ion mode and 923 metabolites in negative ion mode were evaluated in these two groups. After quality control analysis and further volcano plot analysis with FC > 1.5 and FDR < 0.05 using the Student’s t-test, 29 lipidomic metabolites from 4 lipid classes including acylcarnitines, non-alpha-hydroxy-sphingosine ceramides (Cer_NSs), lysophosphatidylcholines (lysoPCs), and lysophosphatidylethanolamines (lysoPEs) were significantly different in migraine patients and controls. Seven of 29 metabolites showed significantly higher levels in migraine patients and 22 exhibited lower levels (Table 3). Of note, Cer_NSs were significantly elevated in migraine patients, and lysoPEs were significantly decreased in migraine patients. Moreover, decreased lysoPE 18:1, lysoPE 18:2 and lysoPE 22:5 were detected in both positive ion mode and negative ion mode. Box plots showed similar results for these lysoPEs in both positive and negative ion mode (Fig. 2). Additionally, except for lysoPC 20:0, other lysoPCs decreased in migraine patients.

**Table 3:** Identification of potential serum lipidomic biomarkers in migraine patients

No.	Metabolites	Ion adduct	Retention time(min)	Experimental Mass,M/z	Fold change (Migraine/Control)	*p* value
Acylcarnitine (positive ion)						
1	Acylcarnitine15:3	[M]+	2.22	380.2813	3.839↑	0.005
2	Acylcarnitine 21:4	[M]+	3.37	462.3571	3.520↑	0.000
3	Acylcarnitine 11:1	[M]+	1.95	328.248	2.094↑	0.004
4	Acylcarnitine 14:3	[M]+	2.01	366.2634	0.478↓	0.007
5	Acylcarnitine 10:1	[M]+	1.9	314.2327	0.465↓	0.001
6	Acylcarnitine 24:0	[M]+	9.93	512.4677	0.435↓	0.009
7	Acylcarnitine 10:0	[M]+	1.95	316.2485	0.402↓	0.002
8	Acylcarnitine 26:0	[M]+	12.49	540.498	0.377↓	0.000
9	Acylcarnitine 20:0	[M]+	6.09	456.4047	0.348↓	0.000
Cer_NS (positive ion)						
10	Cer_NS 34:1; Cer_NS(d18:1/16:0)	[M + H]+	14.75	538.5197	3.520↑	0.000
11	Cer_NS 36:3; Cer_NS(d18:2/18:1)	[M + H]+	13.19	562.5194	2.723↑	0.000
12	Cer_NS 36:2; Cer_NS(d18:2/18:0)	[M + H]+	14.86	564.5356	2.430↑	0.000
lysoPC (positive ion)						
13	lysoPC 20:0; PC(20:0/0:0)	[M + H]+	5.11	552.4031	2.860↑	0.000
14	lysoPC 22:5; PC(22:5(4Z,7Z,10Z,13Z,16Z)/0:0)	[M + H]+	3.06	570.3521	0.474↓	0.002
15	lysoPC 22:0; PC(22:0/0:0)	[M + H]+	8.07	580.4342	0.452↓	0.012
16	lysoPC 18:2; PC(18:2(6Z,9Z)/0:0)	[M + H]+	3.09	520.3403	0.430↓	0.019
17	lysoPC 15:0; PC(15:0/0:0)	[M + H]+	3.14	482.3243	0.423↓	0.000
18	lysoPC 14:0; PC(14:0/0:0)	[M + H]+	2.89	468.309	0.393↓	0.000
19	lysoPC 24:0; PC(24:0/0:0)	[M + H]+	10.33	608.475	0.337↓	0.000
20	lysoPC 16:0; PC(16:0/0:0)	[M + H]+	5.22	496.3397	0.244↓	0.001
lysoPE (positive ion)						
21	lysoPE 18:2; PE(18:2(6Z,9Z)/0:0)	[M + H]+	3.23	478.2933	0.466↓	0.015
22	lysoPE 18:1; PE(18:1(17Z)/0:0)	[M + H]+	3.98	480.3085	0.461↓	0.023
23	lysoPE 22:5; PE(22:5(4Z,7Z,10Z,13Z,16Z)/0:0)	[M + H]+	3.05	528.2969	0.411↓	0.007
24	lysoPE 24:0; PE(24:0/0:0)	[M + H]+	10.62	566.4199	0.349↓	0.016
lysoPE (negative ion)						
25	lysoPE 20:3; PE(20:3(5Z,8Z,11Z)/0:0)	[M-H]-	3.73	502.2946	0.426↓	0.014
	lysoPE 18:1; PE(18:1(17Z)/0:0)	[M-H]-	4.02	478.2952	0.401↓	0.014
	lysoPE 18:2; PE(18:2(6Z,9Z)/0:0)	[M-H]-	3.26	476.2791	0.346↓	0.007
	lysoPE 22:5; PE(22:5(4Z,7Z,10Z,13Z,16Z)/0:0)	[M-H]-	3.27	526.2956	0.388↓	0.014
26	lysoPE 18:3; PE(18:3(6Z,9Z,12Z)/0:0)	[M-H]-	2.89	474.2632	0.380↓	0.002
27	lysoPE 22:6; PE(22:6(4Z,7Z,10Z,13Z,16Z,19Z)/0:0)	[M-H]-	3.12	524.2782	0.338↓	0.003
28	lysoPE 20:4; PE(20:4(5Z,8Z,11Z,14Z)/0:0)	[M-H]-	3.29	500.2796	0.332↓	0.003
29	lysoPE 22:4; PE(22:4(7Z,10Z,13Z,16Z)/0:0)	[M-H]-	3.9	528.311	0.309↓	0.014

### Diagnostic performance of metabolites

A receiver operating characteristic (ROC) curve analysis of potential biomarker levels for differentiating migraine patients is shown in Table 3. The optimal cutoff points as calculated with Youden’s index, sensitivities, specificities, and AUC values are listed. Of these 29 metabolites, 16 showed AUC > 0.80 when migraine patients and controls were compared.

**Fig. 3:**
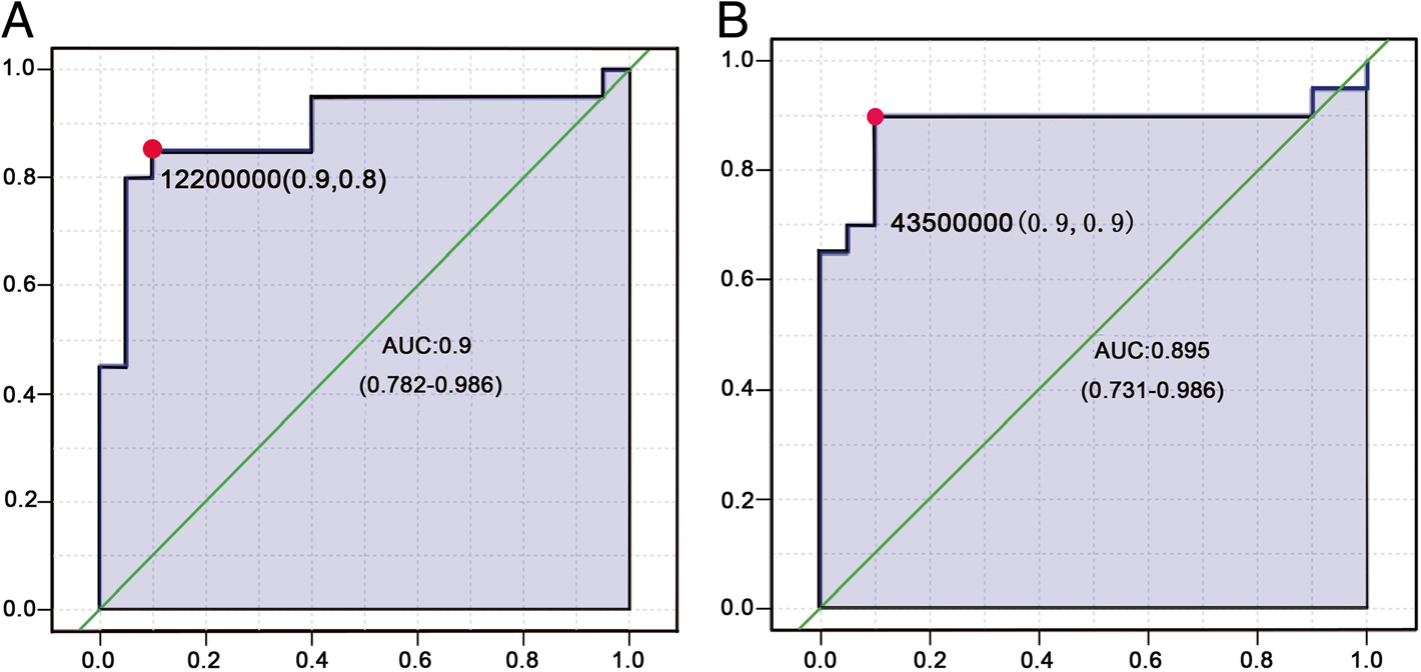
ROC curves for (**a**) lysoPC 16:0; (**b**) lysoPC 20:0 using AUC ≥ 0.9 with a 95% confidence interval for comparing migraine and control groups

The AUC value of lysoPC 16:0 was 0.9 (95% CI = 0.782–0.986), which was the highest of these AUC values, indicating a sensitivity of 90% and specificity of 80% for differentiation of migraine patients and controls. The AUC value of lysoPC 20:0 was 0.895 (95% CI = 0.731–0.986), indicating a sensitivity of 90% and specificity of 90% in these two groups (Fig. 3). LysoPE 18:2, LysoPE 18:1, and lysoPC 22:5 were detected both in positive and negative ion modes, and the AUC values were 0.845 (90% sensitivity and 80% specificity), 0.835 (80% sensitivity and 70% specificity) and 0.802 (80% sensitivity and 80% specificity) respectively (Table 4).

**Table 4:** The AUC, 95% confidence intervals (95% CI), and sensitivity and specificity for ROC curves calculated at the optimal cutoff, together with the *p*-values for 16 significant lipidomic metabolites

No.	Significant Metabolites	AUC	95% CI	Sensitivity	Specificity	Cutoff value	p value
1	lysoPC 16:0	0.9	0.782–0.986	0.9	0.8	1.22 e + 07	0.000
2	lysoPC 20:0	0.895	0.731–0.986	0.9	0.9	4.35 e + 07	0.000
3	Acylcarnitine 21:4	0.888	0.746–0.978	0.8	0.8	1.35 e + 08	0.000
4	Cer_NS 34:1	0.88	0.721–0.965	0.8	0.8	1.97 e + 06	0.000
5	lysoPE 24:0	0.86	0.695–0.96	0.8	0.8	7.13 e + 06	0.000
6	lysoPC 14:0	0.86	0.718–0.949	0.9	0.7	3.56 e + 08	0.000
7	Acylcarnitine 26:0	0.85	0.71–0.954	0.8	0.9	3.84 e + 07	0.000
8	lysoPC 24:0	0.85	0.685–0.976	0.8	0.9	7.27 e + 06	0.000
9	lysoPE 18:2	0.845	0.69–0.958	0.9	0.8	1.18e + 08	0.000
10	lysoPE 18:1	0.835	0.691–0.945	0.8	0.7	4.48 e + 07	0.000
11	lysoPC 22:0	0.83	0.686–0.95	0.8	0.8	1.35 e + 07	0.000
12	lysoPC 15:0	0.828	0.655–0.949	0.8	0.8	3.27 e + 06	0.000
13	Cer_NS 36:2	0.82	0.672–0.941	0.8	0.8	6.36 e + 06	0.000
14	Acylcarnitine 15: 3	0.82	0.674–0.951	0.8	0.8	7.42 e + 06	0.005
15	Acylcarnitine 20: 0	0.805	0.635–0.942	0.8	0.8	6.39 e + 06	0.000
16	lysoPC 22:5	0.802	0.638–0.929	0.8	0.8	1.5 e + 08	0.001

## Discussion

This study examined the lipidomic profiles of migraine patients based on LC-MS. We identified 29 metabolites from 4 lipid classes that were significantly different in migraine patients and controls.

### Acylcarnitine

Acylcarnitines are intermediates in fatty acid and amino acid breakdown generated from the conversion of acyl-CoA species by the action of carnitine palmitoyltransferase 1 (CPT1) [12]. After production of acylcarnitines by CPT1, the mitochondrial inner membrane transporter carnitine acylcarnitine translocase (CACT, or SLC25A20) transports the acylcarnitines into the mitochondrial matrix. Finally, the enzyme CPT2 reconverts acylcarnitines back into free carnitine and long-chain acyl-CoAs [13]. The formation of carnitine conjugates is crucial for the transport of activated fatty acids (acyl-CoAs) from the cytosol across the inner mitochondrial membrane into the matrix where fatty acid oxidation takes place. It has now become clear that a net efflux of acylcarnitine species from the mitochondria into the cytosol and ultimately into plasma is particularly important in situations of impaired fatty acid oxidation for prevention of accumulation of potentially toxic acyl-CoA intermediates in the mitochondrion [9]. Changes in plasma and/or urinary acylcarnitine profiles are used to detect disorders in fatty acid and amino acid oxidation [14, 15]. A broad spectrum of short-, medium-, long- and very long-chain acylcarnitine species is generated from multiple metabolic routes [16].

The current study showed 3 elevated odd-numbered (acylcarnitine 15:3, 21:4, 11:1) and 6 decreased even-numbered forms (14:3, 10:1, 24:0, 10:0, 26:0, 20:0) of medium- to very long-chain acylcarnitines (C10-C26) are found in migraine patients as compared to controls, indicating CPT1 activity for even-numbered acylcarnitine and CPT2 activity for odd-numbered acylcarnitine may be insufficient in these migraine patients. The exact mechanism through which even-numbered acylcarnitine or odd-numbered acylcarnitine might affect migraine remains unknown, and further studies may help determine the role of these acylcarnitines in migraine.

### Cer_NS

Ceramides which are one of the simplest classes of sphingolipids, are lipid signaling molecules which regulate a broad range of both positive and negative cellular functions, including growth, proliferation, motility, adhesion, differentiation, senescence, apoptosis and autophagy [17–20]. Ceramides may be cytotoxic and activate pro-inflammatory cytokines, which promotes oxidative stress and endoplasmic reticulum (ER) stress [21]. Cytotoxic ceramides originating from the liver (or other organs/tissues) which enter the peripheral blood can cross the blood-brain barrier and exert neurotoxic and neurodegenerative effects in the brain [22]. Ceramides are associated with the pathogenesis of Alzheimer’s disease (AD) [20]. Increased plasma levels of very long-chain saturated ceramides (C22:0 and C24:0) were found to be predictive of memory loss and hippocampal atrophy in patients with cognitive impairment [23]. Inflammatory processes are often associated with migraine [24], and high levels of unsaturated very long-chain ceramides (Cer_NS 34:1, Cer_NS 36:3 and Cer_NS 36:2) in these patients may cause activation of pro-inflammatory cytokines, which consequently promote oxidative stress or ER stress and lead to tissue injury. Whether these very long-chain ceramides impair memory or bring about hippocampal injury remains unknown, and further work is needed to clarify the role of these ceramides in migraine.

### LysoPC and LysoPE

LysoPC is a class of phospholipids derived from phosphatidylcholine (PC) which is structural component of cell membranes. LysoPCs are produced by two pathways. The first is the result of partial hydrolysis of PC, so that one of the fatty acids is removed by the action of phospholipase A2 [25]. A second pathway for LysoPC formation occurs by the transfer of one fatty acid of PC to cholesterol by lecithin-cholesterol acyltransferase (LCAT), which is an enzyme that converts free cholesterol into cholesteryl ester. Cholesteryl ester is a more hydrophobic form of cholesterol that is sequestered in the core of lipoprotein particles and in the liver [26]. PE undergoes three successive methylation reactions by PE N-methyl transferase (PEMT) for its full conversion to PC [27].

To date, the relationship between the serum levels of LysoPCs and migraine has not been described. This study showed up-regulation of lysoPC (20:0), and down-regulation of lysoPCs (16:0, 24:0,14:0, 15:0, 22:0, 18:2, 22:5) in migraine patients. The AUC value of lysoPC 20:0 was 0.895 (90% sensitivity and 90% specificity), and the AUC value of lysoPC 16:0 was 0.9 (90% sensitivity and 80% specificity), indicating lysoPC 16:0 and lysoPC 20:0 are potential sensitive and specific biomarkers for migraine. The reason these LysoPCs are altered in migraine is unclear. Oxidative stress, which arises because of an imbalance between the production of reactive oxygen species (ROS) and elimination by antioxidant defense mechanisms has been implicated in migraine [28–31]. Endogenous ROS can cause oxidative damage to DNA, lipids, proteins and extracellular matrix components, including proteoglycans and collagens. Oxidants may also confer susceptibility to other pathogenic processes by disrupting the functions of cytoprotective proteins, such as metabolic enzymes and cell membrane transporters [32]. In this study, most migraine patients suffered from anxiety and insomnia with or without other stimuli. These causes of migraine can lead to oxidative stress. When oxidative stress occurs, the generation of free radicals can activate the phospholipase A2, which hydrolyses PC (PE) to lysoPC (lysoPE). However, at the same time the oxidants disrupt the function of metabolic enzymes such as phospholipase A2, and result in the alterations of lysoPCs and lysoPEs. This mechanism requires further investigation.

LysoPE which is a lyso-type phospholipid, is a metabolic product of phosphatidylethanolamine (PE, a minor constituent of cell membranes) generated by phospholipase A2 [33]. LysoPE has been shown to mobilize intracellular Ca^2+^ through G-protein-coupled receptor (GPCR) in some cells types, such as MDA-MB-231 breast cancer cells, skov3 human ovarian cancer cells and human SH-SY5Y neuroblastoma cells [34–36]. Abnormal synaptic Ca2+ homeostasis and morphology may contribute to chronic neurodegenerative changes in migraine mice [37]. The decreased lysoPEs (18:1, 18:2, 22:5, 18:3, 22:6, 22:4, 20:4, 20:3) maybe lead to abnormal synaptic Ca^2+^ in migraine patients. Exogenous supplementation of these decreased lysoPEs maybe mobilize synaptic Ca^2+^ and be helpful for improving the prevention and treatment of migraine.

## Conclusions

We determined 29 metabolites from 4 lipid classes were significantly different in migraine patients compared to controls: Acylcarnitine, Cer_NS, LysoPC and LysoPE. ROC curve analysis indicated that lysoPC 16:0 and lysoPC 20:0 may be new potential sensitive and specific biomarkers for migraine. We suggest that management of these metabolites may be of benefit in the prevention and treatment of migraine. Further studies should be performed in larger populations of subjects to validate the conclusions of this study.

## Additional file

Additional file 1: The ethics of samples involved in the study. (JPEG 2444 kb)

## Abbreviations

Cer_NS: Non-alpha-hydroxy-sphingosine ceramide
LysoPC: Lysophosphatidylcholine
LysoPE: Lysophosphatidylethanolamine
orthoPLS-DA: Orthogonal partial least squares-discriminant analysis
PCA: Principal component analysis
ROC: Receiver operating characteristic
