# Supplementary figures and images for: Lipidomic analysis of serum samples from migraine patients
Source: Lipids Health Dis. 2018 Feb 2;17:22. doi: 10.1186/s12944-018-0665-0 (PMC5797421; doi:10.1186/s12944-018-0665-0)

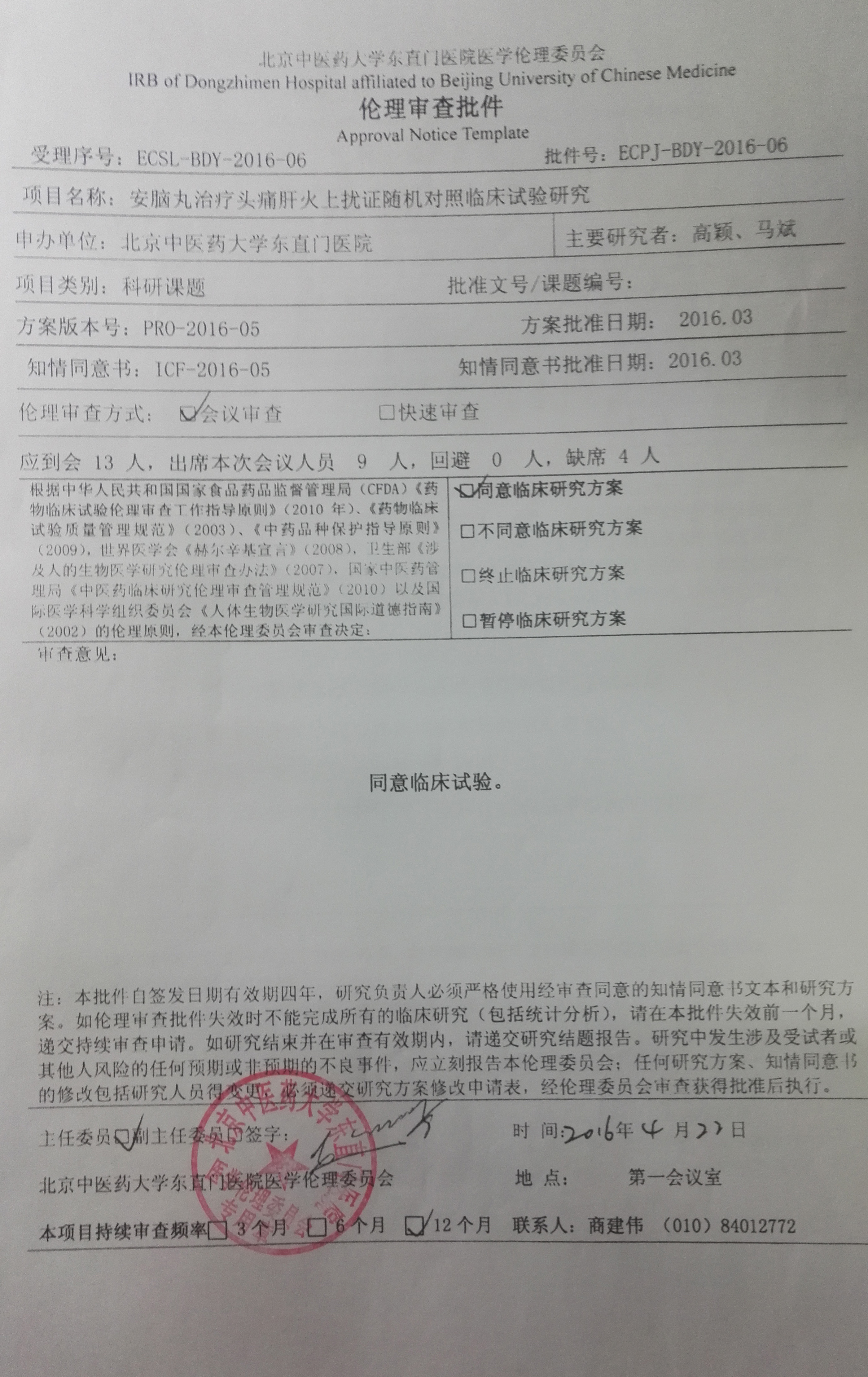

Supplement: Additional file 1: — The ethics of samples involved in the study. (JPEG 2444 kb) [file 12944_2018_665_MOESM1_ESM.jpg]
